# Promoting quality improvement: navigating the conundrum in clinical audit record disclosure

**DOI:** 10.1093/medlaw/fwaf042

**Published:** 2025-12-01

**Authors:** Helen Smith

**Affiliations:** School of Social Sciences, University of Manchester, Manchester, United Kingdom

**Keywords:** clinical audit, disclosure, duty of candour, qualified privilege, quality improvement, safe space

## Abstract

Clinical audit is an essential tool for improving clinical practice by assessing care against evidence-based standards. Yet, the disclosure of clinical audit records raises complex legal and ethical challenges. While transparency fosters patient trust, it also risks discouraging healthcare professionals from full participation due to potential reputational and legal consequences. This article examines the legal frameworks governing clinical audit record disclosure in Ireland, England, and Australia, highlighting key differences in each jurisdiction's approach. Ireland recently introduced legislation that shields clinical audit records from disclosure, whereas Australia’s qualified privilege model provides conditional protection that courts may override in the interests of justice. In contrast, England lacks explicit safeguards except for its newly introduced ‘safe space’ legislation, which applies only to Health Service Safety Investigations Body investigations. Through comparative analysis, this article evaluates the strengths and weaknesses of each approach and advocates for reform in England. It argues for expanding England’s ‘safe space’ model to align more closely with Australia’s qualified privilege framework while addressing its limitations by introducing clearer exceptions and explicit guidance on its interaction with the professional and statutory duties of candour.

## I.INTRODUCTION

This article examines the complex issue of disclosure of clinical audit records and how this problem can and is being addressed by the law. In the aftermath of healthcare scandals such as Mid Staffordshire NHS Foundation Trust, where prolonged abuse and neglect led to hundreds of patient deaths,^1^ or more recently, the inquiry into maternity failings across 14 NHS Trusts in England,^2^ a common question arises: why did these failures persist, and how could such substandard care go unnoticed for so long? These questions spark concern about transparency, accountability, and ethical practices in the healthcare system. Both the professional and statutory duties of candour have been well-discussed in the literature, and arguments in favour of truth-telling have resulted in many jurisdictions introducing a general duty to disclose when harm has occurred. However, little discussion has focused specifically on the disclosure of clinical audit records. Clinical audit, when effectively implemented, plays a crucial role in uncovering deficiencies in clinical practice and potential areas for improvement in patient safety. It can act as a practical tool for identifying problems in clinical practice, often early on, allowing these problems to be addressed before they escalate into prolonged bad practice and controversies. Disclosure of audit results can provide patients with important information that may support their autonomy and ability to make informed decisions about their care. It can also facilitate public oversight of medical practice and provide a useful metric to evaluate whether standards are being adhered to in healthcare institutions.

At the same time, providing patients with access to clinical audit records raises significant concerns for healthcare professionals. There is a risk that practitioners may become reluctant to participate fully and candidly in the audit process if there is a fear of reprisal when records highlight deficiencies in practice. For clinical audit to drive genuine quality improvement for the benefit of the public, clinicians must feel secure in assessing and disclosing shortcomings without apprehension of legal, professional, or reputational harm. This tension creates a fundamental challenge: how can the system foster open and constructive self-assessment to drive quality improvement while simultaneously safeguarding clinicians from the negative consequences of disclosure? If this balance is not carefully managed, there is a risk that audits become superficial exercises, undermining their credibility and effectiveness as tools for quality improvement. The issue of trustworthiness comes to the fore at this juncture. If patients and the public are aware that audit findings are withheld or only selectively disclosed, this may erode their trust in the healthcare system. Conversely, if clinicians believe that their willingness to engage in honest self-evaluation will be penalized, they may disengage from the process altogether. Accordingly, any disclosure strategy should safeguard practitioners to promote their engagement and uphold patient and public trust by ensuring that essential healthcare information is communicated transparently. Striking an appropriate balance between these interests is critical for healthcare institutions to sustain stakeholder trust and to uphold clinical audit as a credible tool for advancing quality of care.

This article contributes to the existing literature on quality assurance activities and clinical governance in hospitals. More specifically, it seeks to enrich the current discourse on clinical audit, such as the work of Berseford and Evans^3^ and Palmer,^4^ by providing a comprehensive analysis of the role of clinical audit in enhancing healthcare quality and the ethical considerations associated with disclosing audit findings. Additionally, the article aims to broaden understanding by undertaking a comparative assessment of the legal safeguards governing the disclosure of clinical audit records in three contrasting legal systems chosen for their differing approaches. Through a comparative analysis of the jurisdictions of Ireland, England, and Australia, the article delves into the benefits and drawbacks of the different legal frameworks, shedding light on the implications for transparency, accountability, and trust in healthcare institutions as well as quality improvement initiatives. The Irish Patient Safety (Notifiable Incidents and Open Disclosure) Act 2023, which recently commenced, protects clinical audit records from disclosure. In contrast, England has little protection for clinical audit records, and records are only protected where they are gathered as part of healthcare investigations under new ‘safe space’ legislation. Australia, on the other hand, strikes a middle ground with its qualified privilege laws, which broadly protect clinical audit records from disclosure; however, this protection can be lifted by the courts in the interests of justice. My analysis advocates for broadening the scope of English ‘safe space’ legislation to better reflect the Australian privilege model. This reform should be paired with explicit guidance to address challenges seen in the Australian approach, such as its relationship with the duties of candour, and to clearly define the exceptions to the legislation’s protections.

## II. CLINICAL AUDIT

The definition of clinical audit, endorsed by the National Institute for Clinical Excellence (NICE) is:

a quality improvement process, that seeks to improve patient care and outcomes through a systematic review of care against explicit criteria, followed by the implementation of change. Aspects of the structure, processes and outcomes of care are selected and systematically evaluated against explicit criteria. Where indicated, changes are implemented at an individual, team, or service level and future monitoring is used to confirm improvement in healthcare delivery.^5^

Clinical audit is designed to enable clinicians and healthcare teams to identify areas for improvement and take decisive action to enhance their practice in alignment with established standards.^6^ The overall aim of an audit is to highlight the discrepancies between the actual practice and standard practice in order to identify the changes that need to be made to improve the quality of care.^7^ Whether conducted at an individual level, within a clinical team, or through national comparisons across healthcare organizations, the overarching objective remains consistent: to elevate standards of care and ensure the highest quality of service within the NHS. Underpinning this imperative is the mandate outlined in the Good Medical Practice guidance by the General Medical Council (GMC), compelling doctors to actively engage in regular reviews and audits to uphold professional standards. As stipulated by the GMC, registered practitioners ‘must take part in regular reviews and audits of your work and your team’s work, this includes taking part in any national audit or outcome review if one is being conducted in your area of practice’.^8^ Further, at an organizational level, the Care Quality Commission’s (CQC) guidance on Health and Social Care Act 2008 regulations mandates that healthcare providers must ‘have systems and processes such as regular audits of the service provided and must assess and monitor and improve the quality and safety of service’.^9^

### A. The historical evolution of clinical audit in England

The origins of clinical audit date back to Florence Nightingale’s pioneering audit assessing mortality rates in a hospital during the Crimean War in 1853.^10^ Following this audit, she was able to improve care on the ward through the purchase of fresh water, fresh fruit, vegetables, and standard hospital equipment and, as a result, the mortality rate reduced from 40 to 2 per cent in less than two years.^11^ In England, the formal integration of clinical audit, initially known as medical audit, began with the 1989 White Paper, *Working for Patients*.^12^ The significance of medical audit as a quality enhancement tool was quickly acknowledged, leading to its inclusion in hospital doctors’ contracts by 1990.^13^ Shortly afterwards, medical audit evolved to embrace a multidisciplinary approach and was termed ‘clinical audit’, defined in 1993 as ‘the systematic analysis of the quality of healthcare, including procedures for diagnosis, treatment and care and the use of resources and resulting outcomes and quality of life for the patient’.^14^

Today, clinical audit is one of six cornerstones of the overall NHS clinical governance framework.^15^ It provides a systematic mechanism through which routine care can be reviewed and is a cyclical process involving: (i) preparation and planning, (ii) measuring level of performance, (iii) making improvements, and (iv) maintaining improvements.^16^ The Health and Social Care Act 2008 forms the statutory basis for the system of registration and regulation which the Care Quality Commission (CQC) operates. The Act mandates that all medical providers (as defined in the Act), including NHS providers, register with the CQC and once registered, the CQC will monitor their compliance with the Health and Social Care Act Regulations.^17^ These regulations set out the duty on providers to ensure that healthcare professionals are enabled to participate in clinical audits to satisfy the demands of the relevant bodies, for example for revalidation and they must regularly assess and monitor the quality of the services provided. They must also use clinical audit findings to ensure that action is taken to protect people who use services from risks associated with unsafe care or treatment and regularly seek the views of service users. The CQC has the power to penalize providers for non-compliance and suspend their registration where appropriate.^18^ The NHS standard contract for 2024/2025 also mandates audit participation through Service Condition 26 (SC26). Providers are required to comply with quality and audit recommendations, and the findings must be available to commissioners. Penalties may apply for breaches of quality standards under SC36, while General Condition 15 (GC15) requires providers to implement clinical audit findings.^19^ Furthermore, the Health Act 2009 and the National Health Service (Quality Accounts) Regulations 2010 provide a statutory basis for the requirement for specific healthcare providers to produce quality accounts, including details of national clinical audit participation and the use of audit reports.^20^ These include clinical audit accounts.

### B. The importance of clinical audit

Despite the growing focus on quality improvement in recent years, a quality gap still exists between ideal care, in line with the best available medical evidence and actual care provided to patients.^21^ While ideal care remains the ultimate objective, standards are established to progress towards this goal, with clinical audit serving as an important tool to assess adherence to these standards and whether they need to be changed. A substantial body of literature has discussed clinical audit’s efficacy in identifying and lessening the quality gap with mixed outcomes as to the results.^22^ This has been attributed to the fact that clinical audits are used to improve specific aspects of health care and can be targeted at different levels, such as organizational level or physician level and rarely affect multiple levels in the same way, making them difficult to compare.^23^ Furthermore, audits are used in different contexts, which makes it difficult to evaluate their effects.^24^ For instance, an audit can be effective in improving one aspect of a department but not another.^25^ For these reasons, measuring clinical audits against one another is not an easy task. However, a systematic review of audit and feedback demonstrated positive overall effects of clinical audit on practice.^26^ Audit is still considered by the NHS to play a fundamental role in improving the quality of clinical practice and patient safety and it ‘offers the greatest potential to assess the quality of care routinely provided for NHS users’.^27^

## III. DISCLOSURE

Quick asserts that no one has a stronger claim to the truth than those who are the direct victims of harm.^28^ However, despite clear ethical underpinnings, the reality is that disclosure to patients is problematic in practice^29^ and not all adverse events are fully and/or honestly disclosed.^30^ While honesty is central to therapeutic relationships, disclosing harm is often seen as a high-risk low reward activity.^31^ Commonly cited barriers to the disclosure of harm to patients include the fear of litigation for doctors and the fear of professional sanctions. Yet, Kelly and Quick in their 2019 paper, examined the history of candour in medicine and their analysis revealed that the reluctance to disclose to patients emerged long before modern liability systems.^32^ They suggest that throughout the history studied,

cumulatively, the medical profession embraced the clinical importance of learning from mistakes and thus favoured intra-professional candour. However, it is also apparent that the profession has not been united on the need for an equivalent approach to patients.

While disclosure in the context of adverse events has been extensively explored in the academic literature, particularly with the introduction of open disclosure laws in a growing number of jurisdictions, the specific issue of disclosure related to clinical audits has received limited attention. This gap in the literature is significant, and this article seeks to address it.

### A. The ethical dilemma

Every day, clinical audits are being carried out in hospitals, seeking to assess and improve the quality of care being given to patients. As an illustration, consider a scenario where an audit on the insertion of PICC lines (peripherally inserted central catheters) reveals a 10 per cent higher occurrence of infection or blood clot complications in a particular hospital than the accepted national standard. This revelation prompts an ethical dilemma concerning the disclosure of these audit results to patients.

On one hand, disclosure upholds the rights of patients to be given the necessary information to exercise their autonomy and make informed decisions about their care. However, that may not be appropriate or desirable as it would likely result in fewer patients opting to have PICC lines inserted in that hospital and may invoke a general sense of fear amongst patients as to why the hospital is underperforming. This could damage a patient’s trust in the healthcare system. Furthermore, as it is inherent to the auditing process that substandard care is identified, this is an opportunity for learning where quality improvement can be brought about, rather than an opportunity to highlight a failure in care or breach of patient rights. Therefore, disclosure to patients or the public of often unpalatable information does not form part of the process, as results are solely used to create statistics for quality improvement.

On the other hand, non-disclosure poses the risk of a future scandal should information on the results of an audit identifying substandard care surface and become public knowledge unexpectedly, causing public alarm and possible consequent legal action, as well as possible reputational damage to the hospital and the doctors working there. The release of such information may slow down quality improvement processes in that hospital due to doctors’ reluctance to take part in an initiative that requires being fully open and honest. Moreover, the question arises as to whether patients have a right to know that they may be more likely to suffer complications at their hospital, so that they can reschedule their care and travel to another hospital for the service if it is available. An integral part of the audit cycle is that substandard care is identified and followed by corrective action. However, in practice, improvement may be partial, take time to implement, or vary in its impact across services. In such circumstances, withholding information during the improvement phase may leave both current and future patients at risk of avoidable harm. A risk of inequality is created here since patients who are unable to travel or those for whom any delay to their care may be detrimental may have less ability to act on such information. However, withholding audit results does not remove this inequality and may in fact worsen it by allowing poorer care to persist for the very patients least able to avoid it.

The above example demonstrates how patients and clinicians may have differing perspectives on the disclosure of clinical audit records. Patients generally seek as much information as possible to make informed choices about their care and to exercise their autonomy. Doctors, on the other hand, while committed to transparency, may be hesitant to disclose certain audit findings if doing so could increase their own professional or legal risk. Beyond the patient–clinician relationship, from the general public’s perspective, there is also a significant public interest in encouraging and maximizing the use of clinical audit to continuously monitor the quality of care being provided in healthcare institutions. The success of the clinical audit as a quality assurance mechanism, therefore, depends on an appropriate balance being struck between all three of these stakeholders’ interests. This necessitates that stakeholders place their trust in healthcare institutions to achieve this balance in a manner that is fair and appropriate for all parties. For this reason, the next section will use institutional trustworthiness as a lens to examine the ethical complexities of clinical audit record disclosure for each stakeholder group.

### B. Institutional trustworthiness and clinical audit record disclosure

Trust is not a luxury but a necessity in health care. Patients are often required to make decisions or comply with treatment plans despite significant epistemic asymmetry,^33^ and they may lack the information, expertise, or contextual knowledge to independently evaluate the safety, quality, or competence of the care they are about to receive. As a result, patients must make judgments under uncertainty, relying not only on individual clinicians but on the broader organizational and systemic structures, and Mechanic observes that ‘our trust in doctors and nurses often generalizes to their organisations and affects our willingness to bring custom to them’.^34^ In this context, trust is a relational response to vulnerability; it emerges from conditions in which one party must place reliance on another with uncertain outcomes.^35^ Medical historian Galeazzi notes that truth-telling is essential to preserving the ‘eternal trust’ in the physician-patient relationship^36^ and research carried out by Edelman in 2022 demonstrates that trust is a key determinant of health.^37^ In discussing trust and its link to trustworthiness, Onora O’Neill posits that ‘trust is only valuable when directed at agents and activities that are trustworthy’.^38^ Trustworthiness has been defined as comprising three key concepts: perceived ability, benevolence, and integrity,^39^ and the trustworthiness of healthcare professionals and healthcare institutions such as hospitals, clinics, and health centres is fundamental to a well-functioning health system in any society.^40^

The way in which healthcare institutions address the disclosure of clinical audit results can profoundly impact their perceived trustworthiness. While audits are primarily designed to be internal tools to support reflective practice, identify systemic weaknesses, and improve future care, they can also reveal patterns of harm, deviations from standard practice, or performance disparities that may be of significance to patients or the public. For trust to be maintained, each stakeholder: patient, practitioner, and the public, must have confidence in their healthcare institution that they will protect their respective interests when considering the merits of disclosure. The next section of the article will consider each stakeholder’s interest through the lens of institutional trustworthiness.

#### 1. Patient interests

While access to external information has become more widespread, patients continue to depend on healthcare professionals and institutions to provide them with comprehensive and accurate information, enabling them to make informed decisions about their care. In their seminal work, ‘Principles of Biomedical Ethics’ Beauchamp and Childress propose four ethical principles: autonomy, beneficence, nonmaleficence, and justice to provide a common moral framework to analyse ethical dilemmas.^41^ Among these principles, autonomy is often considered the most fundamental^42^ and it is defined as ‘at a minimum, self-rule that is free from both controlling influences by others and from certain limitations such as inadequate understanding that prevents meaningful choice’.^43^ This definition of autonomy recognizes that self-rule or decision-making does not have to be absolute but rather, as autonomous as practicable.^44^ It can therefore be best understood as a relational construct that is not absolute but must be maximized, in so far, as is practicable in a given context. In health care, the principle of autonomy suggests that patients should be able to exercise their freedom to make choices about their care and healthcare professionals should respect this.^45^ In this way, the protection of autonomy is largely understood in individualistic terms^46^ and is a right that is in the patient’s interest to protect in so far as possible. For autonomous decisions to be made, patients must possess the necessary level of information they require to make decisions.^47^ This includes information that enables them to assess the risks, quality, and reliability of the care they receive so that they can make an informed decision on their care.

The right of individual patients to information about their care has evolved over the years. This evolution has been reflected by the law, and two key legal principles have emerged in that regard: informed consent and the statutory duty of candour. Informed consent forms the cornerstone of patient autonomy, requiring that patients be provided with sufficient information to make meaningful decisions about their medical care. Determining the necessary standard of information is key to patient trust, and the 2015 *Montgomery v Lanarkshire Health Board* case set the current legal standard for the provision of information.^48^ Ms Montgomery, a small-statured woman with gestational diabetes, was concerned about the risks of vaginal delivery. Her obstetrician did not inform her of the 9–10 per cent risk of shoulder dystocia, a complication more likely in diabetic mothers with larger babies, or the alternative of a caesarean section. During delivery, her baby suffered umbilical cord occlusion due to shoulder dystocia, resulting in cerebral palsy. Ms Montgomery argued that she would have chosen a caesarean section if adequately informed. While the Court of First Instance upheld the *Bolam* test, the UK Supreme Court rejected this approach, holding that the doctor–patient relationship must reflect modern realities, emphasizing patients’ capacity to understand medical information and participate in decisions. The court adopted the test from the Australian High Court’s decision in *Rogers v Whitaker*, which required doctors to disclose material risks relevant to the patient’s concerns.^49^ Importantly, the Court elaborated that the legal test for materiality is twofold and consists of both objective and subjective elements. Under the objective limb, the test for materiality is, whether in the circumstances of the particular case, a reasonable person in the patient’s position would be likely to attach significance to the risk. Under the subjective limb, the doctor was or should be reasonably aware that the particular patient would be likely to attach significance to it.^50^

Applied to the disclosure of clinical audits, this raises the key question: does the legal obligation to disclose information under informed consent extend to these records, and if so, how should such information be communicated to the patient? Unlike routine disclosures about risks of treatment, clinical audits are designed to enhance systemic healthcare quality rather than inform individual patient decisions. Yet, some information within these records, such as higher infection rates for PICC line insertions at a given hospital, may constitute a ‘material risk’ a reasonable patient or that particular patient would consider significant to their treatment decision. This is especially so if the patient has the option to go to another hospital. Similar to *Montgomery* where the risk was 9–10 per cent, if there is a 10 per cent higher risk of infection in PICC line insertion in the hospital, should the patient become aware of this post-infection/post-harm, this would seem to be a violation of the principle set out in *Montgomery* and may have implications for patients trust that they are receiving the requisite information from their doctor. In such circumstances, an individual’s right to make autonomous decisions regarding their future care, along with the necessity for informed consent, may warrant and potentially necessitate the disclosure of clinical audit records to patients.

The realization of patient autonomy is not just reducible to information delivery; patients must also be able to trust that the information delivered is sufficient, relevant, and not selectively withheld. Of particular relevance in this regard are the professional and legal duties of candour. Originally a professional obligation, the duty of candour has since been established as a statutory requirement enshrined in the Health and Social Care Act 2008 (Regulated Activities) Regulations 2014. These duties work alongside each other to require healthcare providers to be transparent and honest with patients, particularly when ‘notifiable patient safety incidents’ occur.^51^ Given the statutory footing of the duty of candour, this article provides a limited discussion of the associated professional duty. The focus is on Regulation 20, which establishes the statutory duty and is discussed in more depth later in this article; however, its relevance here lies in its potential to foster trust by encouraging openness between an institution and the public it serves.^52^ The application of the duty of candour to clinical audit lies in clinical audit’s potential to uncover a ‘notifiable patients safety incident’. For instance, an adverse event that is the subject of a clinical audit could simultaneously trigger an obligation for open disclosure. Similarly, an incident disclosed through the duty of candour might also form part of an ongoing clinical audit. This intersection raises critical questions about the potential impact of open disclosure on clinical audit records and the broader implications for quality assurance activities. If findings from clinical audits uncover incidents that fall within the scope of Regulation 20, and the patient can be identified, the duty of candour requires that this is disclosed to the patient.

It is clear from the above discussion that patients have a fundamental interest in receiving comprehensive and accurate information to enable them to exercise their autonomy and make informed decisions about their care. This right is not merely about the quantity of information disclosed, but also its quality, relevance, and reliability. The law has evolved to protect this right; however, due to significant information asymmetry, patients must still place considerable trust in healthcare institutions to fulfil their legal obligations and provide the information required. To demonstrate trustworthiness, healthcare institutions must demonstrate to patients their commitment to act transparently and in good faith, ensuring that information is not selectively withheld or misrepresented. Accordingly, if clinical audit results are necessary for a patient to be fully informed about their care, these results should be disclosed.

#### 2. Healthcare practitioner interests

Healthcare practitioners occupy a dual role in the clinical audit process, first as the subjects of performance evaluation and secondly, as the agents expected to drive improvement. Participation in audits plays a key role in the operation of a learning healthcare system, defined as a system where researchers, patients, clinicians, and health system leaders work together to identify knowledge gaps, incorporate comparative effectiveness research into clinical practice, and systematically apply the best available evidence.^53^ Yet, healthcare practitioners’ interests in disclosure are shaped by a complex interplay of professional identity, regulatory exposure, and the psychological realities of working in high-risk environments. Consequently, to encourage practitioner participation in clinical audit, they must be able to trust that these interests will be recognized by healthcare institutions.

The doctor–patient relationship is the cornerstone of medical care,^54^ and the protection of this relationship by healthcare institutions is of critical importance to maintaining their trustworthiness. This relationship is fiduciary in nature, grounded in trust, good faith, and confidence, all of which are central to effective medical care.^55^ Haefmeister and Spinos discuss the doctor’s fiduciary duty to disclose, and they note that fiduciaries are charged with a duty of loyalty and must promote the beneficiary’s interest over the fiduciary’s own.^56^ Bartlett argues that these fiduciary characteristics are not merely aspirational ethics but can, and should, have legal force.^57^ Ost offers a narrower perspective.^58^ She argues for legal recognition of fiduciary duties in the context of sexual boundary breaches, where exploitation of vulnerability and informational asymmetry is most acute. Although clinical audit disclosure does not involve exploitation in the sense Ost describes, her analysis reinforces the importance of fiduciary duties and the principle that these duties are designed to prevent professionals from placing their own interests whether reputational, institutional, or defensive above those of the patient. Fiduciary duties demand conduct that sustains interpersonal trust between doctor and patient and healthcare institutions. Transparency in the audit process is an inherent part of fulfilling this obligation. Although doctors may not want audit results disclosed to patients or the public, their fiduciary duties necessitate that they must promote the beneficiary’s interest over their own.

Despite this knowledge of their fiduciary duties, clinicians may be faced with a moral dilemma when considering whether to disclose information. The disclosure of clinical audit records may expose practitioners to potential scrutiny, legal action, and professional repercussions. For instance, in the *Bawa Garba* case, Dr Hadiza Bawa Garba’s reflections regarding the death of 6-year-old Jack Adcock were documented as part of her training requirements. These reflections were not used as evidence against her in court; however, witnesses at her Medical Practitioners Tribunal Service (MPTS) hearing stated that ‘Dr Bawa Garba’s portfolio and reflection were extensively discussed’ at the hearing.^59^ This had widespread implications for practitioners’ trust and since this case, new guidance for doctors recommends that doctors’ reflections should not record identified patient information and should focus on lessons learned rather than specific incidents.^60^ However, like the use of reflections in the *Bawa Garba* case, the use of clinical audit data may be used against a doctor in disciplinary hearings to support an argument that the doctor is not fit to practice. This risk represents a major deterrent to participation in clinical audit as doctors may self-sabotage by participating in a process that identifies and documents their clinical shortcomings. In such an environment, professional trust in the system completely erodes and a climate of fear is created. To promote participation in quality assurance activities, institutions must demonstrate trustworthiness by creating a culture of learning where practitioners can candidly assess their performance without fear of reprisal.

#### 3. Public interest

The emotional, physical, spiritual, and financial vulnerability of patients necessitates trust in healthcare professionals and drives them toward needing to trust numerous organizations and institutions with private information, their health and well-being, and even their lives.^61^ Trustworthiness of the system is necessary to cultivate trust, which is associated with positive health outcomes, adherence to treatment protocols, and effective management of health crises.^62^

When health care is approached as a system rather than an individualized service, the rights-based perspective, which prioritizes individual autonomy, must be weighed against the broader utilitarian imperative to promote the ‘greater good’ of the population. Classical utilitarianism, as articulated by philosophers such as Jeremy Bentham and later, Peter Singer, requires a shift in perspective from individualistic considerations to a universalist stance, urging us to act in ways that maximize overall well-being without disproportionate bias toward ourselves or those immediately connected to us.^63^ This philosophical framework demands that we transcend our immediate sympathies to promote the net good of humanity, or even all sentient beings, as a collective.^64^ In the context of health care, utilitarian ethics emphasize policies and actions that maximize ‘net’ goodness across the population and Kirkwood explains this as directing attention not to an averaged benefit for each individual but to an aggregate improvement in societal welfare.^65^

Applied to clinical audit record disclosure, the value of these records to public must be weighed against the value of transparency to the individual patient. Protecting clinical audit records from disclosure creates a safe environment for practitioners to acknowledge errors, reflect critically on their practices, and collaborate on strategies for improvement without the fear of blame or litigation. However, for this protection to be justifiable to the public and not damage trustworthiness, it must be for the purpose of enhancing the benefits of audit to society and must be balanced against the patient’s interest in disclosure.

The preceding discussion has highlighted the important role that clinical audit plays in quality improvement and its usefulness as an objective quality assurance mechanism. It has also demonstrated that when it comes to the disclosure of clinical audit records, balancing the interests of patients, practitioners, and the public is critical to maintaining each stakeholder’s trust in healthcare institutions. Trustworthiness can be maintained where an appropriate equilibrium is achieved that fosters transparency, safeguards professional integrity, and maintains public confidence in the healthcare system. Building on these considerations, the following section examines how Ireland, England, and Australia have each approached the legal regulation of clinical audit record disclosure and comments on how well each jurisdiction has balanced stakeholder interests.

## IV. LEGAL FRAMEWORKS FOR CLINICAL AUDIT RECORD DISCLOSURE

Different jurisdictions have taken varying approaches to the disclosure of clinical audit records. New landmark legislation has recently been commenced in Ireland, providing for the significant and explicit protection of clinical audit from disclosure, and qualified privilege legislation in Australia protects clinical audit records and other quality improvement activity records from disclosure. Conversely, England has yet to afford clinical audit any explicit legal protection. However, ‘safe space’ legislation has recently been introduced to protect quality improvement information gathered in the course of the Health Services Safety Investigations Board (HSSIB). This section of the article will examine the benefits and potential drawbacks of each jurisdiction’s framework.

### A. The Irish legislation

The Patient Safety (Notifiable Incidents and Open Disclosure) Act 2023 was signed into law in Ireland in April 2023 and commenced on 26 September 2024.^66^ This new Act mandates open disclosure by healthcare providers of certain incidents that occur during the provision of healthcare services.^67^ While a legal structure supporting a professional duty of open disclosure was established in the Civil Liability (Amendment) Act 2017,^68^ the Patient Safety Act strengthens and formalizes these principles, emphasizing the importance of transparency and accountability in health care. Importantly, sections 60 and 61 of the legislation provide explicit protection for clinical audit records, ensuring they are exempt from disclosure under the Freedom of Information Act 2014 and cannot be used as evidence of fault, liability, or in clinical negligence claims. Additionally, these records are safeguarded from invalidating or impacting healthcare practitioners’ insurance and from being used in professional disciplinary proceedings.

This section of the legislation was introduced in response to a recommendation by the Patient Safety and Quality Assurance Commission in the Madden Commission Report on Patient Safety and Quality Assurance in 2008.^69^ This report recommended the introduction of legislation which would give an exemption from the Freedom of Information law and grant legal protection from disclosure of data related to patient safety and quality improvement collected or analysed for internal use or to be shared with others solely for the purpose of safety and quality improvement.^70^ An analysis carried out by the Commission at the time recognized clinical audit as ‘an essential mechanism to monitor quality, assure the safety and quality of services and ensure ongoing quality improvement through identifying opportunities for improvement’.^71^ The critical importance of clinical audit was set out by the Commission in its statement:

Clinical audit arguably constitutes the single most important method which any healthcare organisation can use to understand and ensure the quality of the service that it provides. It is one of the principle methods used to monitor clinical quality and the results provided by clinical audit are a source of indispensable information to patients, the public, clinicians and health managers. It also provides a powerful mechanism for ongoing quality improvement, highlighting incidents where standards are not met and identifying opportunities for improvement.^72^

The Commission also noted the challenges to clinical audit:

If clinicians are to learn and improve from clinical audit, conclusions reached during these processes need to be documented. However, to encourage participation in clinical audit, clinicians need to feel safe with the process and to be assured that it will not be used against them in a punitive manner.^73^

The protective provisions for clinical audit are set out in Part Six of the new Act. The Act defines clinical audit in section 58 as: a clinically led quality improvement process in health care –

- To improve patient care and outcomes through systematic review of care against explicit, specific clinical standards or clinical guidelines and take action to improve care when clinical standards or clinical guidelines are not met and
- which selects aspects of the structure, processes and outcomes of care for systematic evaluation against explicit, specific clinical standards or clinical guidelines.^74^

Section 59 of the Act specifies that its protections apply to clinical audits conducted by healthcare providers or practitioners. It mandates that data collected from these audits must be used solely for improving patient safety and healthcare quality, including sharing aggregated information for this purpose.^75^ This provision ensures that the data is being used to enhance clinical care and benefit patient outcomes. The protections afforded to clinical audit by the Act are set out in sections 60 and 61. Section 60 protects clinical audit records and components of the information provided from disclosure under the Freedom of Information Act 2014 and section 61 provides for three comprehensive protections for clinical audit records:

1. First, section 61(1)(a) information cannot be construed as an admission of fault or liability in any matter arising from the circumstances surrounding the data or clinical audit, including clinical negligence actions.
2. Secondly, section 61(1)(b) prohibits the use of clinical audit information as evidence of fault or liability in court proceedings or clinical negligence actions.
3. Finally, under section 61(1)(c), clinical audit information cannot invalidate or impact the insurance coverage provided for a particular matter. These measures are essential for maintaining the integrity of clinical audit processes and encouraging healthcare providers to engage in quality improvement initiatives without fear of legal repercussions.^76^

Additionally, section 61(2) provides further protection for clinical audit data in professional misconduct proceedings. It stipulates that such data or information cannot be considered an admission of fault, professional misconduct, poor professional performance, or unfitness to practise by a health practitioner.^77^ This section explicitly states that clinical audit data is not admissible as evidence of fault or misconduct in proceedings related to professional disciplinary matters.

This recently introduced legislation provides significant protection to doctors participating in clinical audits. While the legislation introduces mandatory open disclosure and encourages transparency in medical practice, a paradox arises in its provisions on clinical audit. Clinical audit is afforded substantial protection from disclosure under the same Act and notably, no provision for this protection to be lifted by a third party, such as the court, is included in the Act. As highlighted by its title, the Act’s emphasis on open disclosure demonstrates its symbolic importance in fostering public trust. Yet, it includes provisions that exempt clinical audit from open disclosure obligations without oversight from the court. This raises concerns about the coherency of the legislation’s goals, and from the public’s perspective, this dual standard could undermine the perceived legitimacy of the Act and erode confidence in its commitment to transparency.

That being said, the extensive protection afforded to clinical audit within the Act reflects the critical role it plays in quality improvement. By safeguarding clinical audit, the legislation underlines both the importance of supporting clinicians engaged in these audits and the government’s broader commitment to enhancing patient safety and care quality. In this context, the protection of clinical audit aligns with the Act’s overall aim of improving patient safety. However, it is essential that the public is informed of this goal and educated on the benefits of clinical audit to justify the paternalistic protections and preserve public trust.

Further, concerns have been raised about the narrowness of the definition of clinical audit in the Act. For instance, an Irish national audit of surgical mortality proposed to improve practice and reduce surgical mortality through systemic peer review will not go ahead due to it falling outside of this definition. There have been calls to extend legal protection to include other quality assurance activities. However, the Department of Health commented that

from a policy perspective, without a clear and discrete definition of quality improvement activities, concerns could arise for the public about too wide an application of extensive legal protections and their alignment with the principle and spirit of open disclosure.^78^

As the Act has very recently been commenced, it is too early to comment on how effective the current definition is or will be and whether it should be expanded. Careful monitoring of the effectiveness of this legislation in encouraging participation in clinical audit will be necessary to determine whether the definition should be expanded.

### B. The English legislation

In England, there is currently no dedicated legislation in place to safeguard clinical audit records from disclosure. Instead, healthcare professionals are required by law and by professional standards to uphold a duty of candour, and patients have the right to request information about themselves through freedom of information and data protection laws. Recently, there has been a notable development in English law with the introduction of section 120 of the Health and Care Act 2022, which introduces a prohibition on the disclosure of information gathered in the course of HSSIB investigations. While this legislation specifically applies to HSSIB investigations, its emergence suggests a potential shift towards protecting quality assurance activities, including clinical audits, in the future.

The professional duty of candour was introduced into the GMC Code of Conduct in 1998, which noted that if a patient suffered serious harm, professionals ‘should act immediately to put matters right…explain fully to the patient what has happened…[and] when appropriate you should offer an apology’.^79^ The GMC updated its Guidance for 2001–2011, adding a section entitled ‘Being Open and Honest with Patients if things go wrong’.^80^ This extended the duty to cases where patients experienced harm or distress, stating that an apology and explanation should be provided. Current GMC Guidance named ‘Openness and honesty when things go wrong: The professional duty of candour’ sets out the professional duty of candour and includes a section entitled ‘Being open and honest with patients in your care, and those close to them, when things go wrong.’ This section provides guidance on the need to discuss risks with patients, the circumstances where apologies are necessary, when to speak to the patient or those close to them, what to do if they do not want to know details and saying sorry and how to be open and honest about near misses.^81^ The statutory duty of candour, on the other hand, was introduced in 2014 in Regulation 20 of the Health and Social Care Act 2008.^82^ The duty comprises two key components: mandating that registered persons ‘act openly and transparently with relevant persons in relation to care and treatment provided’, and requiring providers to report ‘notifiable safety incidents’, ‘as soon as reasonably practicable’ offering support to affected patients.^83^ A ‘notifiable safety incident’ for a health service body refers to an unexpected incident during the provision of a regulated activity that, in the opinion of a healthcare professional, could result in the death of the service user where the death is directly linked to the incident rather than to the natural progression of the user’s illness or condition, or severe, moderate, or prolonged psychological harm to the service user.^84^ For any other registered person, a ‘notifiable safety incident’ means an unintended incident that, in the opinion of a healthcare professional, could result in the death of the service user, where the death is directly linked to the incident, impairment of sensory, motor, or intellectual functions lasting 28 days or more, prolonged pain or psychological harm, shortened life expectancy, or the need for treatment to prevent the death or serious injury of the service user.^85^ This notification must:

- be given in person by one or more representatives of the registered person,
- provide an account, which to the best of the registered person’s knowledge is true, of all the facts the registered person knows about the incident as at the date of the notification,
- advise the relevant person what further enquiries into the incident the registered person believes are appropriate,
- include an apology, and
- be recorded in a written record which is kept securely by the registered person.^86^

The legislation does not explicitly define what information must be disclosed to patients during the factual account provided by the representative. Consequently, clinical audit records identifying systemic failings that may have contributed to or caused a ‘notifiable patient safety incident’ could be subject to disclosure. While this lack of definition promotes flexibility and aligns with the principles of transparency, it also creates ambiguity about the scope of disclosure.

Studdert and Richardson, in their analysis of quality assurance activities and the duty of candour in Australia, highlight that quality assurance activities may intersect with open disclosure when they produce findings relevant to unexpected outcomes of care.^87^ The overlap between clinical audits and open disclosure lies in the types of information they generate and the purposes they serve. Open disclosure is fundamentally about sharing information with patients regarding incidents affecting their care, including what happened, why it happened, and what measures will be taken to prevent recurrence. While clinical audits are primarily designed as quality improvement tools for assessing whether standards of care are being met, they may indirectly reveal systemic failings or patterns of substandard care. These findings could, in certain cases, be relevant to understanding the causes of a patient safety incident. For example, the results of a clinical audit might show that certain processes or standards were not adhered to, which could be seen as contributing factors to an adverse event. In such cases, the findings of the audit could become relevant to the open disclosure discussion, particularly when transparency about systemic issues is necessary to fulfil the duty of candour. However, the nature of the clinical audit as a broader evaluative tool (rather than an investigation into specific incidents) complicates its direct applicability to open disclosure requirements. Thus, while clinical audit findings are not explicitly mandated for disclosure, they are not protected from it either. There is a grey area where their relevance to a specific incident may justify or even necessitate disclosure under the duty of candour. Without protection or guidance as to how to navigate clinical audit record disclosure, healthcare providers may be obligated to disclose records as part of their factual account.

Patients can also access information held by the NHS or other organizations through a Freedom of Information (FOI) request under Part 1 section 8 of the Freedom of Information Act 2000. They can also access their own personal information through a Subject Access Request (SAR) under Article 15 of the UK General Data Protection Regulations (GDPR). The right of access under FOI applies only to information held by public authorities, including NHS bodies, and does not extend to the private sector. By contrast, the right of access under SARs applies to any organization, public or private, that holds an individual’s personal data.^88^ Part 1 of the FOI Act gives the public the right to request any recorded information held by a public authority, including NHS bodies.^89^ However, FOI requests only apply to non-personal information. This means individuals cannot use FOI requests to access their own medical records or any confidential patient data. Instead, access to personal health information is governed by SARs under GDPR and the Data Protection Act 2018.^90^ Under this legislation, individuals are entitled to obtain personal data held about them, subject to certain exceptions. These include disclosures that are likely to harm the individual or another person, records relating to a third party, and records containing medically harmful information, such as a terminal diagnosis that the patient does not know about yet.^91^ Access to clinical audit records, particularly those held within the National Clinical Audit and Patient Outcomes Programme (NCAPOP), follows a structured data access process. The Healthcare Quality Improvement Partnership (HQIP**)**, which commissions NCAPOP audits, manages access to these records. To obtain audit data, individuals or organizations must submit a Data Access Request Form to the Data Access Request Group (DARG). DARG, in collaboration with the relevant audit provider, assesses applications based on factors such as ethical considerations, compliance with data protection laws, and the intended use of the data before granting approval.^92^

A significant legislative protection for quality assurance activity known as ‘safe space’ legislation has been introduced in England in s.122 of the Health and Care Act 2022. Originally contained in the National Health Service Trust Development Authority (Healthcare Safety Investigation Branch) Directions 2016, the ‘safe space’ legislation is based on the principle that

contributions that are comprehensive and candid are more likely to be made where they may be made in the confidence that they will be used not for the purposes of apportioning blame or establishing liability but for the purposes of identifying improvements or areas for improvement if any which may be made in patient safety.^93^

The 2016 directions were replaced by section 122 of the Health and Care Act 2022. Although this legislation does not explicitly use the term ’safe space’, section 122 establishes a prohibition on the disclosure of materials obtained during specified health service inquiries.^94^ Effective as of April 2023, this enactment also established the Health Services Safety Investigation Body (HSSIB), which replaced the Healthcare Safety Investigations Branch (HSIB) and is tasked with conducting investigations into incidents that have occurred during the provision of health care services and have or may have implications for patient safety.^95^ There are a number of exceptions to this prohibition on disclosure, which are set out in section 123. These include: the exceptions set out in Schedule 14 of the Act, where disclosure is required by any other provision of this part of the Act and regulations made by the Secretary of State. Schedule 14 sets out the following exceptions to the prohibition on disclosure: disclosure for purposes of investigations, where the Chief Investigator believes it to be reasonably necessary for the purposes of carrying out the HSSIB investigation function, where it is necessary for the prosecution or investigation of an offence under section 121, disclosures relating to safety risks, and disclosure by the High Court or to exercise the Chief Investigator’s function.

The case of *Turner v Sheffield Teaching Hospitals NHS Foundation Trust* provides an example of where the court will intervene and lift the legal privilege provided and order disclosure, although this case took place prior to the introduction of the new legislation, when the 2016 Directions were still in place.^96^ In this case, the Claimant was severely injured, and the circumstances of the injury were investigated by the Third Party (the HSIB). During the course of the proceedings, the Claimant brought an application for specific disclosure against the Third Party for the midwifery interview transcripts provided to the HSIB during the investigation. The NHS resisted the application on the basis that the documents were not under their control and the Third Party resisted the application on the basis that the disclosure would undermine their investigations.^97^ The Court held that in this case, the transcripts were ‘central to the issue to be decided’ and permitted the disclosure.^98^

This ability for the High Court to order disclosure has now been formally recognized in Schedule 14 of the new legislation, and it will be interesting to see in future cases how the court applies this exception. The new legislation formalizes legal privilege or 'safe space’ protections for information gathered during healthcare investigations, which may include clinical audit records. The prohibition on disclosing this information is so strong that it constitutes a criminal offence for anyone working for the HSSIB, or who has ceased to be connected with the HSSIB, to knowingly or recklessly disclose such information to another person, highlighting the commitment to enforcing this prohibition and safeguarding the information.

Already, there have been calls to make ‘safe space’ records public. Rob Behrens, chair of the Parliamentary and Health Service Ombudsman office is calling for ‘safe space’ material to be available to other investigators. He opines that the idea of guaranteeing a ‘safe space’ threatens his role of protecting patient safety and providing access to justice for families. ‘Effective access to information is vital to give the ombudsman the facts it needs to assess the complaints brought to it,’ he told the BMJ. ‘The HSSIB safe space will leave my team and me without knowledge of serious medical failings.’^99^ However, a ‘safe space’ is not guaranteed by this legislation as there are exceptions, including provisions allowing the Court to lift the protection. Jean McHale criticizes this, noting that for NHS outsiders such as patients and their families, going to court to lift the protection is challenging and significantly more costly and concludes that ‘this is a regrettable move and one which work against trust and transparency’.^100^ Academic Oliver Quick has also argued firmly against the introduction of the statutory ‘safe space’, suggesting that it is ‘wrong in principle and problematic in practice’.^101^ His principal objection is that it is ‘morally wrong to exclude those most affected by the incident from accessing all available information’. Quick’s objection is grounded in the belief that safe spaces may dilute the duty of candour, potentially obscuring the full explanation of incidents and impeding the process of genuine accountability. This argument appears particularly persuasive given that incidents investigated by the HSSIB are likely to also qualify as ‘notifiable safety incidents’, which would ordinarily trigger the duty of candour. Consequently, legislation that shields information about such incidents seems to contradict the very essence of this duty and, as Quick highlights, significantly dilutes its intended impact. Similarly, Peter Walsh in his Editorial ‘Safe Space’ or ‘Secret Space’? Proposals for Safety Investigations in England’ argues that the introduction of the safe space would ‘create a mockery of the Duty of Candour and existing guidance’ and would be ‘deeply harmful to the relationship of trust that should exist between patients/families and health professionals and the NHS’..^102^ No guidance has been published on the legislation’s interaction with the duty of candour and these concerns are valid and have been left unanswered. As observed by Jean McHale ‘candour and safe space are, as we have seen, very uncomfortable bedfellows’.^103^

A review of the duty of candour has recently been announced by the Department of Health and Social Care. As part of this, a review of the duty’s interaction with safe space legislation should be conducted to provide guidance and clarity to the public and to healthcare professionals on the interaction between the two. Guidance should also be provided on what information must be disclosed in open disclosure conversations. In this context, the Australian experience, where the relationship between qualified privilege laws and the duty of candour has already been explored by an expert governmental working group, offers a particularly useful point of comparison for English legislation.

### C. The Australian legislation

In Australia, certain quality assurance activities, such as clinical audit, are formally protected by legislation. In 1992, qualified privilege legislation was introduced in Australia at the federal level in an amendment to the Health Insurance Act 1973 (Part VC). It was originally introduced to facilitate open and frank discussion by health professionals participating in peer review of clinical cases by hospital and health service committees.^104^ The legislation aimed to ‘provide a very important protection or immunity to enable these activities to take place with a recognition of the importance of confidentiality’.^105^ By preventing the release of identifiable information, including that of patients and healthcare professionals, the scheme shields participants from potential embarrassment, damage to their reputation, or legal ramifications.^106^

To obtain qualified privilege under this scheme, organizations conducting eligible quality assurance activities of national or multijurisdictional significance, including healthcare service providers, educational and research bodies, medical colleges, health professional associations, and government bodies must submit an application. This applies only to organizations whose quality assurance activities take place in publicly funded health services involving Australian government funding, including Medicare benefits, public hospital services, health programme grants, or prescribing pharmaceutical products under the Pharmaceutical Benefits Scheme. The health service will then receive a declaration that they fall under the qualified privilege scheme, and this is effective for a 5-year-period after which it may be renewed.^107^ Statutory support for qualified privilege is provided under Part VC of the Health Insurance Act 1973^108^ and Part 10 sections 84–89 of the Health Insurance Regulations 2018.^109^ This federal scheme operates alongside and does not override state and territory regimes, and all states and territories except the Northern Territory have similar qualified privilege legislation.

A statutory duty of candour has recently been introduced in one state in Australia, Victoria. The Australian Council for Safety and Quality in Health Care introduced the National Standard for Open Disclosure in 2003, and following a review of the Standard in 2011, it was replaced by the Open Disclosure Framework endorsed nationally in 2013.^110^ This framework introduced a professional duty of candour and the Victorian state government is the first state in Australia to formalize a statutory duty of candour. Prior to this, the Victorian Government’s expert working group on open disclosure raised significant concerns regarding the interaction between qualified privilege laws and open disclosure obligations. The group highlighted widespread confusion among healthcare professionals and organizations about the scope and application of privilege laws and recommended a review of Victoria’s qualified privilege regime due to inconsistent approaches to releasing information.^111^ They also noted instances where protections were misused or unjustifiably invoked to withhold critical information, including from health complaints processes.^112^ Ten years prior to this, Studdert and Richardson in their review of qualified privilege and open disclosure argued that the connection between state qualified privilege laws and open disclosure processes is relatively weak however, the corollary of this weak connection is that qualified privilege laws do not provide robust protection over the content of open disclosure conversations. Therefore, when the two intersect, for instance when a clinical audit reveals consistent substandard care and this substandard care led to harm, a grey area exists as to what information is protected by qualified privilege and what can be disclosed to the patient. Despite, the introduction of the statutory duty of candour in Victoria and recommendations by the working group, no further guidance has been provided as to how qualified privilege interacts with open disclosure. Given that the duty now carries legal weight in the state of Victoria, this uncertainty is likely to heighten concerns among healthcare professionals, making them more defensive in sharing information when they are unsure, potentially to the detriment of patients.

Ahern, Hopper, and Loh’s 2019 paper also comments on the lack of clarity surrounding when qualified privilege can be lifted this time in relation to the court’s power to lift the protection. They note that while qualified privilege offers a measure of protection to healthcare professionals, Australian case law allows confidentiality and privacy statutes to be overridden in the public interest.^113^ Courts retain discretion to weaken such protections, even when qualified privilege is invoked. Therefore, they argue that qualified privilege should be viewed as a defense rather than conferring absolute immunity under the Health Insurance Act.^114^ However, with the shift towards patient-centred care, it becomes increasingly difficult to justify absolute immunity under the law, as this tilts the balance excessively toward paternalism. The judiciary’s ability to lift the protection offers vital checks and balances, ensuring that the privilege can be set aside when the interests of justice demand it. Labelling qualified privilege as a ‘defence’ implies that the default position is one of non-protection, which is not the case, as most quality assurance information will be protected under the legislation and will not go before the court. However, clarity is necessary as to when the court will intervene, and this stands in contrast to the safe space legislation, where the same statutes that establish protections also clearly delineate exceptions. In Australia, the absence of equivalent statutory clarity introduces significant uncertainty regarding when and under what circumstances the protection might be removed. Ahern, Hopper, and Loh argue that the current framework does not adequately balance safeguarding professional interests with addressing the public’s right to transparency, calling for a comprehensive review of the legislation. Their call for review was also echoed by the working group and by Studdert and Richardson. While the legislation provides provisions aimed at improving the quality of care, its lack of specificity appears to be undermining the realization of this objective. A review is necessary to provide clarity on key issues, including the legislation’s interaction with the new statutory duty of candour and the circumstances in which the court will lift the protections.

## V. COMPARING THE APPROACHES

The new Irish legislation has specifically designated clinical audit as exempt from open disclosure requirements, demonstrating the important role clinical audit plays in improving standards of care and the high value that policymakers place on its protection. From an ethical standpoint, this protection seems to take a utilitarian point of view that favours the value of clinical audit records to the healthcare system rather than the individual patient’s right to open disclosure in every aspect of their care. This approach can be justified by the principle that enhancing the overall quality of care within the healthcare system ultimately benefits individual patients, as improvements at a systemic level tend to permeate down to patient care over time. By providing extensive protection to clinical audit records, the Irish legislation offers reassurance to health professionals, encouraging their full participation and engagement without fear of reprisal. This symbolizes a move away from ‘blame culture’ towards a more ‘just culture’, encouraging learning from mistakes and continuous improvement in clinical practice. The introduction of this new Irish legislation should strengthen clinical audit practices; however, it also raises considerable concerns about accountability.

Transparency involves conducting affairs in the open and being subject to public scrutiny.^115^ In a large public organization such as the Health Service Executive (HSE) in Ireland, public scrutiny serves an important role in holding the healthcare system accountable when failures occur, serving as a critical mechanism to uncover shortcomings. While the protection of clinical audit is undeniably important, the Irish legislation may go too far, as it lacks adequate checks and balances, effectively blocking public oversight and failing to provide a mechanism for judicial oversight. Unlike the English ‘safe space’ legislation or the Australian qualified privilege framework, the Irish provisions do not include an explicit mechanism for lifting the protection by court order. This creates an exceptionally strong safeguard, crossing the line into paternalism. This rigidity prevents access to information that might be critical to patients or families, potentially undermining the principle of accountability.

In stark contrast, England offers minimal protection for clinical audit records outside of healthcare investigations, exposing them to potential disclosure to the public and use in clinical negligence cases and disciplinary proceedings. The lack of protection means that health providers may have to disclose clinical audit findings as part of open disclosure conversations with patients when providing a factual account of what happened and what is being done to prevent recurrence. Such susceptibility to disclosure is likely to deter doctors’ participation in audits due to concerns about potential reprisal. England’s ‘safe space’ legislation partially addresses this concern, offering protections from disclosure but only to information gathered during HSSIB investigations and providing a mechanism for the court to lift the protections. Expanding this legislation to include all clinical audit records, irrespective of their purpose, while preserving exceptions for necessary disclosures, would achieve a balanced and coherent approach. Such an approach would safeguard healthcare professionals’ participation in quality assurance activities while maintaining accountability in cases where justice requires transparency.

Currently, the English legislation for ‘safe spaces’ only protects quality improvement activity after there has been a clinical failure. It focuses on reacting to incidents rather than preemptively preventing them. Although reactive investigations provide valuable lessons to prevent future harm, this approach fails to protect continuous quality improvement activities, leaving doctors exposed. Implementing continuous protection for clinical audit records represents a more coherent and equitable approach. Shielding crucial information only during investigations appears arbitrary and disproportionately unfair to those most in need of answers when serious harm has already occurred and at a point when the duty of candour has been triggered. If this legislation is expanded, it is first necessary to address concerns about its interaction with the duty of candour. A comprehensive review is essential and must be accompanied by detailed guidance to clearly delineate the boundaries between the two. This will ensure that healthcare professionals are confident in what information can or cannot be shared during open disclosure meetings.

In this regard, the Australian qualified privilege regime offers a valuable model for England to learn from. Unlike the English model, Australian legislation provides continuous protection, extending beyond healthcare investigations to encompass ongoing quality improvement activities. This broader protection facilitates a more proactive approach to identifying substandard care and fostering a culture of accountability and continuous improvement. By safeguarding quality assurance processes, Australian legislation minimizes the risk of a blame culture while enabling systemic enhancements to patient safety. An important feature of the Australian regime is its ability to lift protections from disclosure by court order in the public interest. However, in practice, the lack of clarity surrounding when the court will lift the protection seems to be potentially hindering the legislation’s effectiveness. Due to uncertainty about when the privilege will be lifted, healthcare professionals cannot carry out quality assurance activities without fear of disclosure. Consequently, the legislation falls short of achieving its intended aim.

A review of the legislation is essential to establish clear exceptions to the protections it provides. The revised framework should be accompanied by comprehensive guidance outlining the factors courts will consider when determining whether to lift the privilege. This guidance must emphasize that such protections will only be lifted in truly exceptional circumstances and provide a list of factors that will be considered in this decision, ensuring clarity and fostering confidence among healthcare professionals. Further, the interaction between the duty of candour and qualified privilege needs to be considered, particularly in the state of Victoria, where there is a statutory duty of candour. It is essential that the status of clinical audit records or other quality assurance records at open disclosure meetings is clarified.

## VI. THE CASE FOR REFORM

England should draw on the Australian experience by expanding its protections to enhance coherence while proactively addressing the potential conflict between the legislative protections and the duty of candour at an early stage. Like Australia, this policy should be implemented for quality assurance activities carried out in publicly funded health service providers. This approach reflects the fact that these providers are accountable to taxpayers and Parliament, already operate under statutory transparency regimes such as the Freedom of Information Act 2000 and public reporting requirements, and bear a corresponding obligation to maintain public trust. The private sector, especially wholly private providers operating outside the NHS framework, lacks equivalent systemic oversight, and extending protections to them may erode public confidence. A review can later be undertaken as to whether this should apply to the private sector to create one unified framework.

Once an organization’s application for qualified privilege has been approved, all quality assurance activities by that organizations are then covered regardless of the type of medical treatment, and this applies to both national and local clinical audit. This breadth avoids fragmenting the protection and ensures coherence in encouraging candour across the audit cycle. To ensure the effectiveness of these reforms, a comprehensive review of both the duty of candour and the safe space legislation is essential. This review must provide detailed guidance for healthcare professionals and the public, clearly defining the circumstances under which clinical audit records or other quality assurance materials can be shared during open disclosure meetings. This guidance should also clarify the conditions under which protections may be lifted, emphasizing that such instances will remain truly exceptional.

## VII. CONCLUSION

This article has sought to illuminate the critical importance of clinical audit to the healthcare system and the challenge faced by policymakers in deciding whether clinical audit records should be disclosed to the public in the interests of transparency. Despite its significance, this area has received limited attention in England, where the current protections for clinical audit records are confined to HSSIB investigations. This article has put forward the case for reform, advocating for a more coherent legislative framework that extends protections to clinical audit records beyond these investigations. Such reforms would address gaps in the current system, ensuring that clinical audits can fulfil their potential to drive improvements in care without undermining the duty of candour or public confidence. By learning from international experiences, particularly Australia, England has the opportunity to craft legislation that balances the need for confidentiality with the principles of transparency and accountability. This approach would not only enhance participation in clinical audits but also strengthen the overall quality assurance mechanisms within the healthcare system.

## Footnotes

1 Mid Staffordshire NHS Foundation Trust Public Inquiry, *Report of the Mid-Staffordshire NHS Foundation Trust Public Inquiry* HC 898-1 (The Stationary Office 2013).

2 GOV.UK, ‘14 NHS Trusts the Focus of National Maternity Investigation’ (GOV.UK, 15 September 2025) <https://www.gov.uk/government/news/14-nhs-trusts-the-focus-of-national-maternity-investigation> accessed 15 October 2025.

3 Nigel Beresford and Timothy Evans, ‘Legal Safeguards for the Audit Process’ [1999] 319 British Medical Journal 654.

4 Claire Palmer, ‘Clinical Governance: Breathing New Life into Clinical Audit’ [2002] 8 Advances in Psychiatric Treatment 470.

5 Ross Scrivner and Claire Morrell, *Principles for Best Practice in Clinical Audit* (Radcliffe Medical Press 2002) 1.

6 Robin Burgess and John Moorhead, *New Principles of Best Practice in Clinical Audit* (Radcliffe Publishing Ltd 2011) xi.

7 Pasquale Esposito and Antonio Dal Canton, ‘Clinical Audit, a Valuable Tool to Improve Quality of Care: General Methodology and Applications in Nephrology’ [2016] 3 World Journal of Nephrology 249.

8 General Medical Council, ‘Guidance on Supporting Information for Revalidation’ (GMC, 31 January 2024) <https://www.gmc-uk.org/registration-and-licensing/managing-your-registration/revalidation/guidance-on-supporting-information-for-revalidation/quality-improvement-activity#:∼:text=You%20must%20take%20part%20in , are%20unable%20to%20participate%20directly> accessed 03 January 2024.

9 Health and Social Care Act (Regulated Activities) Regulations 2014, Regulation 17.

10 The Regulation and Quality Improvement Authority, ‘A Brief History of Clinical Audit’ <https://www.rqia.org.uk/RQIA/files/2e/2e14bc09-0c9f-4480-9ff7-30cebea5f608.pdf> accessed 01 January 2024

11 Hosein Karimi and Negin Masoudi Alavi, ‘Florence Nightengale: The Mother of Nursing’ [2015] 4 Nursing and Midwifery Studies 1.

12 Home Office, *Working for Patients: Medical Audit Working Paper* (Cmd 555, 1989).

13 ibid.

14 ibid.

15 Claire Palmer, ‘Clinical Governance: Breathing New Life into Clinical Audit’ (2002) 8 Advances in Psychiatric Treatment 470.

16 Margaret Hughes, ‘*Clinical Audit: A Manual for Lay Members of the Clinical Audit Team’* (Health Quality Improvement Partnership 2012).

17 Health and Social Care Act (Regulated Activities) Regulations 2014.

18 Health and Social Care Act (Regulated Activities) Regulations 2014, reg 23.

19 NHS England, ‘2024/2025 Standard Contract’ (NHS, 16 March 2023) <https://www.england.nhs.uk/publication/full-length-nhs-standard-contract-2023-24-particulars-service-conditions-general-conditions/> accessed 12 March 2024.

20 Health Act 2009, s 8 and National Health Service (Quality Accounts) Regulations 2010, sch.

21 Kathryn McDonald, Ellen Schultz and Christine Chang, ‘Closing the Quality Gap: Revisiting the State of Science’ (2013) AHRQ Publication No 12(13) EO17 <https://www.ahrq.gov/research/findings/evidence-based-reports/gapsumtp.html> accessed 22 April 2024.

22 Joanne lord and Peter Littlejohns, ‘Evaluating Healthcare Policies: A Case for Clinical Audit’ (1997) 315 BMJ Publishing Group Ltd 668.

23 Lisanne Hut-Mossel and others, ‘Understanding How and Why Audits Work: A Protocol for a Realist Review of Audit Programmes to Improve Hospital Care’ [2021] 7 PLoS One 1.

24 ibid.

25 ibid.

26 Noah Ivers and others, ‘Audit and Feedback: Effects on Professional Practice and Healthcare Outcomes’ (2012) 13 Cochrane Database of Systematic Reviews 1.

27 Scrivner and Morrell (n 5).

28 Oliver Quick, ‘Duties of Candour in Healthcare: The Truth, the Whole Truth and Nothing but the Truth?’ (2022) 30 MLR 324.

29 Catherine Kelly and Oliver Quick, ‘The Legal Duty of Candour in Healthcare: the lessons of history?’ (2019) 70 Northern Ireland Legal Quarterly 77.

30 BJ Myren and others, ‘Disclosing Adverse Events in Clinical Practice’ (2022) 37 Health Communication 191.

31 Quick (n 28).

32 Kelly and Quick (n 29) .

33 Rebecca Brown, ‘Deferring to Expertise whilst Maintaining Autonomy’ (2024) Episteme 1–20 <https://doi.org/10.1017/epi.2024.3> accessed 05 August 2025.

34 David Mechanic, ‘Changing Medical Organisation and the Erosion of Trust’ (1996) 74 The Milbank Quarterly 171.

35 Carole Robinson, ‘Trust, Health Care Relationships, and Chronic Illness’ (2016) 3 Global Qualitative Nursing Research 1.

36 Olivio Galeazzi, ‘Truth, Disease and Prognosis: An Historical-Anthropological Analysis’ (2006) 809 Annals of the New York Academy of Sciences 40.

37 ‘Edelman, 2022 Edelman Trust Barometer Special Report: Trust and Health (August 2022) <https://www.edelman.com/sites/g/files/aatuss191/files/2022-08/2022%20Trust%20Barometer%20Special%20Report%20Trust%20and%20Health%20with%20Talk%20Track.pdf> accessed 31 July 2025.

38 Onora O’Neill, ‘Linking Trust to Trustworthiness’ (2018) 26 International Journal of Philosophical Studies 293.

39 Roger Mayer, James Davis and David Schoorman, ‘An Integrative Model of Organizational Trust’ (1995) 20(3) Academy of Management Review 709.

40 Samantha Meyer and others, ‘Trust in the Health System: An Analysis and Extension of the Social Theories of Giddens and Luhmann’ (2008) 17 Health Sociology Review 177, 178.

41 Tom Beachamp and James Childress, *Principles of Biomedical Ethics* (OUP 2019).

42 Immanuel Kant and others, *Groundwork of the Metaphysic of Morals - A New Translation* (OUP 2019).

43 Beachamp and Childress (n 41) 99. .

44 ibid.

45 Michelle Gold, ‘Is Honesty Always the Best Policy? Ethical Aspects of Truth-telling’ (2004) 34 Internal Medicine Journal 578.

46 Nahide Basri, ‘Coherence of the Principle of Patient Autonomy in the English Medical Law: A Re-evaluation’ (2020) 5 LSE Law Review 31.

47
*Montgomery v Lanarkshire Health Board* [2015] UKSC 11.

48 ibid.

49
*Rogers v Whitaker* (1992) 175 CLR 479.

50
*Montgomery v Lanarkshire Health Board* [2015] UKSC 11, 87.

51 Health and Social Care Act 2008 (Regulated Activities) Regulations 2014, reg 20.

52 Simon Gardiner, Douglas Morrison and Simon Robinson, ‘Integrity in Public Life: Reflections on a Duty of Candour’ (2021) 24 Public Integrity 217.

53 Jonathan Casey and others, ‘What Can a Learning Healthcare System Teach Us about Improving Outcomes’ (2021) 27 Current Opinion in Critical Care 527.

54 Antonio Yaghy, ‘The Cornerstone of Medical Care’ (2023) 14 Canadian Medical Education Journal 135.

55 Ama Edwin, ‘Non-Disclosure of Medical Errors and Egregious Violation of Ethical Principles’ (2009) 43 Ghana Medical Journal 34.

56 Thomas Hafemeister and Selina Spinos, ‘Lean on Me: A Physician’s Fiduciary Duty to disclose an emergent Medical Risk to the Patient’ (2009) 86 Washington Law Review 1167.

57 Peter Bartlett, ‘Doctors as Fiduciaries: Equitable Regulation of the Doctor–Patient Relationship’ (1997) 5 Medical Law Review 193.

58 Suzanne Ost, ‘Breaching the Sexual Boundaries in the Doctor–Patient Relationship: Should English Law Recognise Fiduciary Duties?’ (2016) 24 Medical Law Review 206.

59 Michael Gorton AM and Matthew Goessler, ‘Can Trainee’s Personal Reflection be Used in Court? (Revisited)’ (RusselKennedy.com, 17 May 2021) < https://www.russellkennedy.com.au/insights-events/insights/can-trainees-personal-reflections-be-used-in-court-revisited> accessed 20 February 2024.

60 Clare Dyer, ‘Doctor’s Personal Reflections Should Not Include Case Details’ (2018) 362 British Medical Journal 3890.

61 John Platt and Susan Dorr Goold, ‘Betraying, Earning, or Justifying Trust in Health Organizations (2023) 53 Hastings Center Report s53.

62 Elisavet Souvatzi and others, ‘Trust in Healthcare, Medical Mistrust, and Health Outcomes in Times of Health Crisis: A Narrative Review’ (2024) 14 269 Societies 1.

63 Henry Sidgwick, *The Methods of Ethics* (7th edn, Hackett Publishing 1981).

64 Peter Singer, *Practical Ethics* (CUP 1979).

65 Ken Kirkwood, ‘In the Name of the Greater Good?’ (2010) 2 Emerging Health Threats Journal 7092.

66 Patient Safety (Notifiable Incidents and Open Disclosure) Act 2023.

67 Oireachtas, Patient Safety (Notifiable Incidents and Open Disclosure) Act 2023 (Oireachtas, 2 May 2023) < https://www.oireachtas.ie/en/bills/bill/2019/100/> accessed 05 April 2024.

68 Civil Liability (Amendment) Act 2017.

69 Department of Health & Children, *Building a Culture of Patient Safety: Report of the Commission on Patient Safety and Quality Assurance* (Brunswick Press 2008).

70 Catherine Reilly, ‘Audit of Surgical Mortality will Not Progress’ (*Medical Independent*, 25 June 2023) < https://www.medicalindependent.ie/in-the-news/audit-of-surgical-mortality-will-not-progress/> accessed 20 April 2024.

71 Department of Health, *Revised General Scheme of the Health Information and Patient Safety*: Regulatory Impact Analysis <https://assets.gov.ie/11441/ce1d167063254135a89e72aa7a96c728.pdf> accessed 04 April 2024.

72 ibid.

73 ibid.

74 Patient Safety (Notifiable Incidents and Open Disclosure) Act 2023, s 58.

75 ibid s 59.

76 ibid, s 61.

77 ibid, s 61(2).

78 ibid.

79 GMC, Good Medical Practice (GMC 1998) paras 16–18.

80 GMC, Good Medical Practice (GMC 2006) para 30.

81 GMC, *Openness and Honesty When Things Go Wrong: The Professional Duty of Candour* (GMC 2015) paras 7–22.

82 Health and Social Care Act 2008 (Regulated Activities) Regulations 2014, reg 20.

83 ibid.

84 ibid, reg 20(7).

85 ibid, reg 20(7).

86 ibid, reg 20(3).

87 David Studdert and Mark Richardson, ‘Legal Aspects of Open Disclosure: A Review of Australian Law’ (2010) 193 Medical Journal of Australia 273.

88 BMA, ‘Access to Health Records Guidance for Health Professionals in the United Kingdom’ (*BMA*, May 2018) <https://www.bma.org.uk/media/1868/bma-access-to-health-records-nov-19.pdf> accessed 12 April 2024.

89 NHS Resolution, ‘Freedom of Information’ (*NHS Resolution*, 7 February 2023) <https://resolution.nhs.uk/freedom-of-information/#:∼:text=The%20Freedom%20of%20Information%20Act,promote%20greater%20openness%20and%20accountability> accessed 12 April 2024.

90 Data Protection Act 2018.

91 NHS, ‘Good Practice Guidelines for GP Electronic Records’ (*NHS*, 10 February 2023) <https://www.england.nhs.uk/long-read/subject-access-requests-sar/> accessed 14 April 2024.

92 HQIP, ‘Data Access at HQIP’ < https://www.hqip.org.uk/national-programmes/accessing-ncapop-data/> accessed 10 December 2024.

93 The National Health Service Trust Development Authority (Healthcare Safety Investigations Branch) Directions 2016, 6(1)b.

94 Health and Care Act 2022, s 122.

95 ibid, s 110(1).

96
*Turner v Sheffield Teaching Hospitals NHS Foundation Trust* [2023] EWHC 3452 (KB).

97 ibid.

98 ibid.

99 Jane Feinman, ‘Should Safe Space Material Be Kept Confidential?’ (*BMJ*, 20 September 2022) <https://www-bmj-com.manchester.idm.oclc.org/content/378/bmj.o1944> accessed 20 April 2024.

100 Jean McHale, ‘Patient Safety, Safe Space and the Duty of Candour: Reconciling the Irreconcilable’ in John Tingle Clayton O’Neill and Morgan Shimwell (eds), *Global Patient Safety Law Policy and Practice* (Routledge 2018), 96.

101 Quick (n 28).

102 Peter Walsh, ‘‘Safe Space’ or ‘Secret Space’? Proposals for Safety Investigations in England’ (2017) 22 Clinical Risk 81.

103 McHale (n 100).

104 Susannah Ahern, Ingrid Hopper and Erwin Loh, ‘Qualified Privilege Legislation to Support Clinical Quality Assurance: Balancing Professional and Public Interests’ (2019) 20 Medical Journal of Australia 343.

105 ibid.

106 Department of Health and Aged Care, ‘About Commonwealth Qualified Privilege Scheme’ (*Health.gov*, 7 February 2024) <https://www.health.gov.au/our-work/commonwealth-qualified-privilege-scheme/about#why-the-scheme-is-important> accessed 29 February 2024.

107 ibid.

108 Health Insurance Act 1973, Part VC.

109 Health Insurance Regulations 2018, Part 10 (84–89).

110 Australian Commission on Safety and Quality in Health Care, ‘The Australian Open Disclosure Framework’ (ACSQHC, Sydney 2013) <https://www.safetyandquality.gov.au/sites/default/files/migrated/Australian-Open-Disclosure-Framework-Feb-2014.pdf> accessed 10 August 2025.

111 Expert Working Group to advise on legislative reforms arising from Targeting Zero, ‘A Statutory Duty of Candour Report to the Minister for Health’ (*Victoria State Government*, August 2020) <https://www2.health.vic.gov.au/hospitals-and-health-services/quality-safety-service/better-safer-care/statutory-duty-of-candour> accessed 10 August 2025.

112 Studdert and Richardson (n 87).

113 Susannah Ahern, Ingrid Hopper and Erwin Loh, (n 104).

114 Department of Health and Aged Care, ‘About Commonwealth Qualified Privilege Scheme’ (*Health.gov*, 7 February 2024) <https://www.health.gov.au/our-work/commonwealth-qualified-privilege-scheme/about#why-the-scheme-is-important> accessed 29 February 2024.

115 Siri Wiig and others, ‘Transparency in Health Care: Disclosing Adverse Events to the Public’ in Mathilde Bourrier and Corinne Bieder (eds), *Risk Communication for the Future Towards Smart Risk Governance and Safety Management* (SpringerOpen 2018) 111–125.

